# *Acanthodasys caribbeanensis* sp. n., a new species of Thaumastodermatidae (Gastrotricha, Macrodasyida) from Belize and Panama

**DOI:** 10.3897/zookeys.61.552

**Published:** 2010-10-11

**Authors:** Rick Hochberg, Sarah Atherton

**Affiliations:** University of Massachusetts Lowell, One University Avenue, Lowell, MA 01854, 01.978.934.2885

**Keywords:** Meiofauna, Caribbean, Panama, Belize, gastrotrich, taxonomy

## Abstract

We describe one new species of Acanthodasys (Gastrotricha, Macrodasyida, Thaumastodermatidae) collected from sublittoral sites around Carrie Bow Cay, Belize and Isla Colón in the Bocas del Toro archipelago, Panama. Though eight species of Acanthodasys are currently recognized, no species has yet been reported from the Caribbean. Acanthodasys caribbeanensis **sp. n.** is characterized by the lack of lateral adhesive tubes, the presence of ventrolateral adhesive tubes, and with cuticular armature in the form of both spineless and spined scales. The spineless scales are not elliptical as in other species of Acanthodasys, but are instead variable in shape and closely resemble the spineless scales of species of Diplodasys. Spined scales bear uniancres up to 50 µm long and are the largest reported in the genus. Uniancres are arranged dorsally around the mouth rim and distributed in five distinguishable columns. Adult size varies from 325–625 µm long.

## Introduction

The phylum Gastrotricha contains some of the smallest (0.1mm-3mm) benthic marine invertebrates. In general, marine gastrotrichs are restricted to the interstitial pore spaces between sand grains, making them overlooked in most tropical biodiversity investigations. Knowledge of gastrotrich biodiversity in the Tropical Northwestern Atlantic (aka wider Caribbean) is almost entirely limited to species lists or descriptions of novel taxa from only a handful of sites in the five Caribbean ecoregions: the Gulf of Mexico (Todaro 1994; Todaro et al. 1995), South Florida ecoregion (Thane-Fenchel 1970; Schoepfer-Sterrer 1974; Decho et. al. 1985; Evans and Hummon 1991; Evans 1994), Bahamanian ecoregion (Renaud-Debyser 1963), Lesser Antilles ecoregion (Kisielewski 1984; Hummon 2010) and Central Caribbean ecoregion including Colombia (Hummon 1974) and Panama (Hochberg 2008). Additional unpublished records of marine gastrotrich diversity and biogeography can be found in Hummon (2009).

Here, we document a new species of Acanthodasys (Macrodasyida, Thaumastodermatidae) from two Central Caribbean islands, Carrie Bow Cay on the Belizean barrier reef complex and Isla Colón in the Bocas del Toro archipelago, Panama. Investigations on both islands were conducted as part of a Smithsonian sponsored biodiversity project to barcode the local marine meiofauna.

## Methods

Gastrotrichs were collected from a variety of sublittoral sites around Carrie Bow Cay, Belize (Jan.13–28, 2010) and Bocas del Toro, Panama (June 6–19, 2010). Research was performed at the Smithsonian field station on Carrie Bow Cay, Belize and Smithsonian Tropical Research Institute, Bocas del Toro, Panama, respectively. Sediments were collected by SCUBA in bags and buckets and brought back to the field stations for subsampling. Extraction of gastrotrichs was performed with the following protocol: 1) approximately 100 cm^3^ of sediment was combined with ca. 900 cm^3^ of 7.5% MgCl_2_ in a 1 L Erlenmeyer flask and allowed to rest for 15 mins; 2) the flask was gently shaken and the supernatant was decanted over a 48 µm mesh; and 3) the mesh was gently washed with seawater into a petri dish. Specimens were sorted with a Leica EZ4 stereomicroscope, transferred to a glass slide, and viewed with a Zeiss A1 compound microscope equipped with DIC (differential interference contrast). Light micrographs were captured with a Sony Handycam digital camera. Measurements of individual specimens were performed with an ocular micrometer. Lengths and positions of organ systems are described in terms of percentage body units, where total body length from anterior (U00) to posterior (U100) is 100 units.

Specimens were prepared for museum archival using the following protocol: fix in 2.5% gultaraldehyde in 0.1M phosphate buffer saline (PBS; pH 7.4) for at least one week; rinse with PBS for 1 hr; expose to 1% OsO_4_ in 0.1M PBS for 1 min (to increase constrast); rinse in PBS for 15 min; dehdryate through an ethanol series; transferr to propylene oxide for 30 min; and embed in epon on a glass microscope slide (coverslipped and placed in an oven at 60ºC for 24 hrs). Type specimens are deposited in the National Museum of Natural History, Smithsonian Institution, Washington, DC.

Abbreviations: PIJ, pharyngeointestinal junction; TbA, anterior adhesive tubes below mouth rim; TbL, lateral adhesive tubes; TbP, posterior adhesive tubes on caudal pedicles; TbVL, ventrolateral adhesive tubes.

## Results

Order Macrodasyida Remane, 1925 [Rao and Clausen 1970]

Family Thaumastodermatidae Remane, 1927

Subfamily Diplodasyinae Ruppert, 1978

Genus Acanthodasys Remane, 1927

### Acanthodasys caribbeanensis sp. n.

urn:lsid:zoobank.org:act:2ED007EB-D7F8-4E0D-9DC4-37BAFA49B247

Figs 1 3 

#### Type locality.

Station CBC10.19, Carrie Bow Cay Reef, coarse sand patches on ridge, 15 m depth, 16°48.127N, 88°04.607W. Sediments collected by Cheryl Thacker on January 23, 2010.

#### Holotype.

Adult specimen, reproductively mature, 625 µm long, resin preparation: specimen in dorsoventral orientation. USNM #1145897

#### Other localities.

Station BRS2010-104, Wild Cane Rock, Bocas del Toro, Panama, 14 m depth, medium coarse sand plain, 9°21.016N, 82°10.335W. Sediment collected by Daniel Gouge on June 8, 2010.

#### Paratypes.

Cat. No. USNM 1145898, Adult specimen, minimum 425 µm long (curled), resin preparation: specimen on side. Cat. No. USNM 1145899, Adult specimen, minimum 450 µm long (curled), resin preparation: specimen in dorsoventral orientation.

#### Material examined.

Ten specimens. Three prepared for archival.

**Figure 1.**
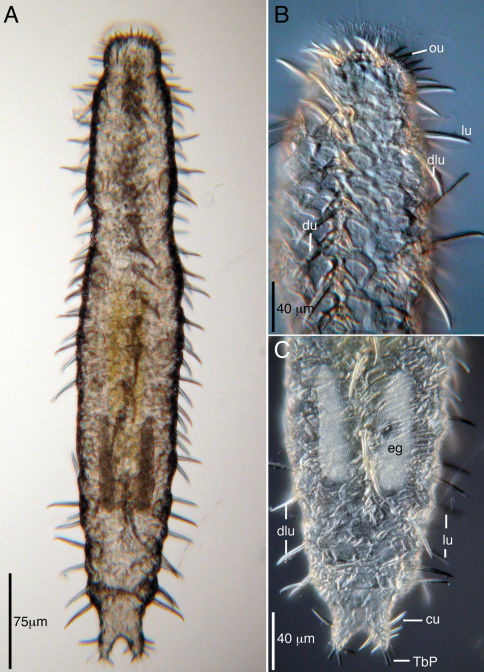
Acanthodasys caribbeanensis sp. n. from Carrie Bow Cay, Belize **A** Dorsal view, transmitted light **B** Closeup of anterior end, dorsal view, DIC optics **C** Closeup of posterior end, dorsal view, DIC optics. Abbreviatons: cu caudal uniancres, du dorsal uniancre, dlu dorsolateral uniancre, eg egg, lu lateral uniancre, ou oral unianre, TbP posterior adhesive tubes.

#### Diagnosis.

Acanthodasys with an adult body length from 375 µm to 625 µm long. Maximum body width at mouth/PIJ/midpoint of body is 30/62/100 µm. Pharynx to 238 µm long. Cuticle of spineless and spined scales. Spineless scales of various shape. Spined scales bear uniancres to 50 µm long, distributed as one dorsal column flanked by two dorsolateral columns and two lateral columns. Uniancres also extend dorsally across mouth rim. Ten epidermal glands per side. Up to nine TbA per side inserting directly on body surface at mouth rim. TbL absent. At least 43 TbVL per side beginning at PIJ and extending onto the outer edges of the caudal lobes. Two TbP insert terminally on caudal lobes, two insert medially. Hermaphroditic, with paired testes and single glandular caudal organ. Rosette organ on left dorsolateral side of body; single ovary present.

#### Etymology.

Named after the region of origin, referring to the Caribbean (Latin adjectival ending: *ensis*; *caribbeanensis*).

#### Description.

**Figure 2.**
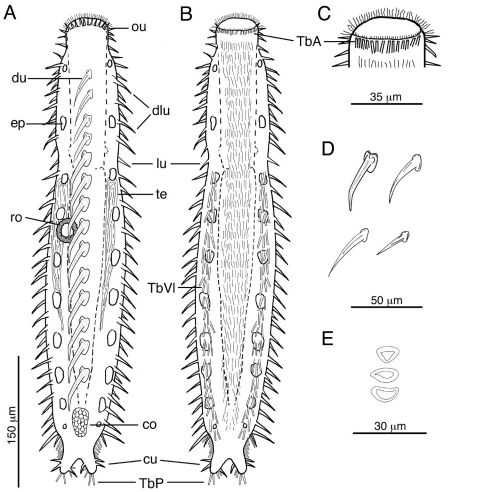
Acanthodasys caribbeanensis sp. n. **A** Habitus, drawn in dorsal view. **B** Habitus, drawn in ventral view. **C** Closeup of head region showing the distribution of TbA **D** Examples of different shapes of uniancres on the body **E** Examples of different scale shapes on dorsal and ventral sides. Abbreviatons: co caudal organ, cu caudal uniancres, dlu dorsolateral uniancre, du dorsal uniancre, ep epidermal gland, lu lateral uniancre, ou oral unianre, ro rosette organ, TbA anterior adhesive tube, TbP posterior adhesive tubes, TbVl ventrolateral adhesive tubes, te testis.

The description is based on the holotype (adult, 625 µm long), with ranges given from specimens measured in vivo. Body strap-shaped and 425–625 µm long (Fig. 1A). Mouth region narrow; body inflates at U04, narrows again at U08, and widens toward the trunk. Widths of mouth at U04/pharynx region at U08/PIJ at U36/trunk at U50/caudal base at U92 are 30/67/105/125/20 µm. Pharynx 238 µm long with pharyngeal pores at U32. Dorsal rim of oral hood, ca. 10 µm wide, free of cuticular spines and fringed with numerous mobile cilia and few stiff sensory hairs to 10 µm long. Epidermal glands to 18 µm diameter and to 10 per side. Ventral locomotory cilia present as a complete field beginning at ca. U08 and extending to the caudum (Fig. 2B).

#### Cuticular armature.

**Figure 3.**
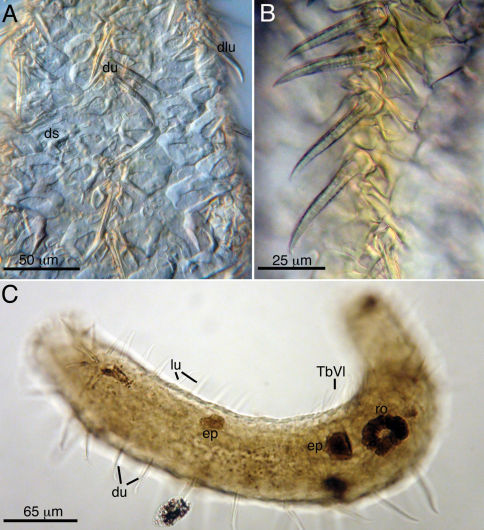
Acanthodasys caribbeanensis sp. n. from Carrie Bow Cay, Belize (A, B) and Bocas del Toro, Panama (C) **A** Closeup of dorsal cuticle showing dorsal uniancres, dorsolateral uniancres and spineless dorsal scales **B** Closeup of dorsal uiancres **C** Paratype (Cat. No. USNM 1145899) of Panamanian specimen showing the rosette organ (gland). Abbreviations: du dorsal uniancres, ep epidermal gland, lu lateral uniancre, ro rosette organ, TbVL ventrolateral adhesive tubes.

Cuticular spines evident at low magnification and arranged around the periphery of the head, along the trunk and on the caudal lobes (Figs. 1, 2). Spined- and spineless scales present. Approximately 10–12 oral uniancres line the dorsal periphery of the head behind the “naked” region of the oral hood (Fig. 1B). Uninacres slightly staggered in position; uniancres increase in size from medial position (10 µm long) to a lateral position along head (U06, 23 µm long). Uniancres in lateral and dorsolateral positions continue along the trunk as individual columns. The trunk contains a total of five columns of uniancres: one dorsal, two dorsolateral, and two lateral columns (Figs. 1A, 2A). Sixteen uniancres in dorsal column begin at U08 and extend to U80, increasing in size from 20–50 µm long down the length of the body, and with a slight bend or posterior curvature at the apex (Fig. 3B). Dorsolateral columns of ca. 14–16 spines that increase in length along the trunk, from 17–35 µm long and with a slight posterior curvature or distinguishable bend (Fig. 2D); column ends at U85. Lateral columns of 23–26 spines that increase in length along the trunk, from 20–35 µm, and mostly with a straight shape but oriented in a slight posterior direction; column ends at U87. Five small uniancres present on each caudal lobe, 8–20 µm long, in somewhat dorsolateral position (Fig. 1C). Cuticle between uniancres present as spineless scales. Scales of various shape; most dorsal scales have an obtuse triangular shape with the apex pointed posteriorly; some ventral scales same shape as dorsal scales; others of varying shapes (Figs. 1B, 2E, 3A). Center of each scale with a triangular or eye-shaped depression. Scales ca. 15 µm wide and arranged in 5 columns on dorsal surface; ventral surface also with scales but the number of columns was undetermined.

#### Adhesive tubes.

Anterior adhesive tubes (TbA), 8 per side to 12 µm long, distributed along the mouth margin in a single row (Fig. 2B,C). TbL are absent. TbVL arranged in bilateral columns, to 23 µm long, begin at PIJ and extend posteriorly on to the lateral edge of the caudal lobes; tubes on caudal lobe edges each ca. 15 µm long (Fig. 2B). TbP distributed on caudal lobes as two terminal tubes to 16 µm long and two medial tubes on the inner edge of the caudal lobes to 12 µm long.

#### Digestive tract.

Mouth terminal to 30 µm wide. Pharynx to 238 µm long with pharyngeal pores at base around U32. Intestine narrow and tapering toward posterior end. Anus at U88.

#### Reproductive system.

Hermaphroditic, with paired testes at PIJ. Vasa deferentia extend posteriorly, but their point of termination was not observed. Glandular caudal organ, ca. 30 µm long, at U90 (Fig. 2A). Rosette organ, ca. 27 µm diameter, present at U42 on left, dorsolateral surface. Glands of the rosette organ stain intensely with OsO_4_ (Fig. 3C). Single egg present, ca. 57 µm x 100 µm (Fig. 1C).

## Taxonomic remarks

At present, there are eight valid species of Acanthodasys described globally: Acanthodasys aculeatus Remane, 1927; Acanthodasys algarvensis Hummon, 2008 (see Hummon and Todaro 2010); Acanthodasys arcassonensis Kisielewski, 1987; Acanthodasys carolinensis Hummon, 2008; Acanthodasys fibrosus Clausen, 2004; Acanthodasys flabellicaudus Hummon & Todaro, 2009; Acanthodasys lineatus Clausen, 2000; and Acanthodasys silvulus Evans, 1992. Five species mentioned by Ruppert (1978) are not described and considered nomina nuda (Kisielewski 1987; Hummon 2008). Among described species, Acanthodasys aculeatus, the type species, has the widest geographic distribution with conspecifics reported from the coasts of Europe (Kisielewski 1987) and the Mediterranean and Black Seas (reviewed in Todaro et al. 2003) to as far south as the coastlines of India (Naidu and Rao 2004), the Maldive Islands (Gerlach 1961), and the Atlantic coast of Florida, USA (Evans 1992). Hummon (2009) has reported Acanthodasys aculeatus from a variety of other locales. However, to date, no species of Acanthodasys are reported from the Caribbean.

Among the eight described species, only Acanthodasys aculeatus Remane, 1927 and Acanthodasys arcassonensis Kisielewski, 1987 possess spineless scales distributed among their uniacres as found in Acanthodasys caribbeanensis sp. n. However, the new species differs from both described species in several significant ways: the head profile differs between Acanthodasys caribbeanensis sp. n. and Acanthodasys arcassonensis; the uniancres are arranged in distinguishable columns in the new species (Figs 1, 2); the uniancres are larger in Acanthodasys caribbeanensis sp. nov (up to 50 µm long) compared to all other species (e.g, Acanthodasys arcassonensis to 11 µm long; Acanthodasys fiborosus to 20 µm); the spineless scales are not elliptical in the new species but instead variously shaped; and the new species possesses adhesive tubes in a ventrolateral (TbVL) as opposed to a lateral (TbL) position. This latter characteristic is noteworthy because the arrangement of TbVL of Acanthodasys carribeanensis sp. n. is more similar to the arrangement observed in species of Diplodasys, the sister taxon of Acanthodasys within the Diplodasyinae (Ruppert 1978), than to other species of Acanthodasys (e.g., compare to Acanthodasys flabellicaudus Hummon & Todaro, 2009 and Diplodasys sanctimariae Hummon & Todaro, 2009).

Interestingly, there are several aspects of the cuticle of Acanthodasys caribbeanensis sp. n. that appear unique and worth further mentioning. First, the uniancres of the new species are the largest described spines (see Fig 1A; up to 50 µm long on an adult specimen 625 µm long) in the genus and the largest spines known from any species of Macrodasyida. Second, the spineless scales of Acanthodasys caribbeanensis sp. n. are more similar in appearance to the spineless scales of species of Diplodasys than they are to the scales descibed for other species of Acanthodasys. For example, the scales of Diplodasys minor Remane, 1936 have a similar shape to those of Acanthodasys caribbeanensis sp. n., and several species have scales with a wide depressed region in their center like the scales of Acanthodasys caribbeanensis (e.g., see Diplodasys ankeli ssp. pacifica Schmidt, 1974; comparison of species’ scales in Kisielewski 1987). Note, however, that the scales of Acanthodasys caribbeanensis sp. n. appear to lack any obvious ribbing or texture (Fig. 3A) that often characterizes the scales of species of Diplodasys.

Another peculiar feature of Acanthodasys caribbeanensis sp. n. is the relatively wide size distribution of reproductive adults. We found specimens ranging from 325 µm long in Panama to 625 µm long in Belize, and all specimens showed clear evidence of reproductive maturity i.e., paired testes and/or a solitary rosette organ were present (Fig. 3C). According to Ruppert (1978), the rosette organ is a glandular ring around the female gonopore, but whether it is present in specimens prior to the maturation of female gametes is not mentioned. According to our surveys, specimens of Acanthodasys caribbeanensis sp. n. may have a rosette organ but no obvious ova or testes. As evidence, we have prepared a paratype (Cat. No. USNM 1145898) of a smaller adult specimen, ca. 400 µm long (slightly curled), with a clear rosette organ but without evidence of eggs or testes. Unfortunately, the rosette organ can be difficult to see in live specimens, but becomes apparent if animals are fixed briefly in OsO_4_ (see methods); the OsO_4_ greatly enhances the contrast of many tissues including the epidermal glands (see Fig. 3C).

## Supplementary Material

XML Treatment for Acanthodasys caribbeanensis
